# In Memoriam: Georg K. Uhlschmid (1937–2023)

**DOI:** 10.3389/ti.2023.11492

**Published:** 2023-05-19

**Authors:** Rudolf Steiner

**Affiliations:** University of Zurich, Zürich, Switzerland

**Keywords:** obituary, transplantation, immunology, ESOT, angiogenesis, biopolymers

Georg K. Uhlschmid was born in Graz (Austria) on 13 July 1937. He started his medical education at the Karl-Franzens-University Graz in 1956 after leaving the classical gymnasium with distinction and became Dr. med. univ. (*doctor medicinae universae)* in 1962. His early formation years as general surgeon were spent in Linz and Basel from 1963–1966. Prof. Rudolf Nissen attested him “an excellent clinical and operative talent combined with an agreeable, tactful way towards his patients, being greatly appreciated by his colleagues and nursing staff”. Supported by a private fellowship he then worked at the Institute of Reconstructive Plastic Surgery, New York University Medical Center, in the transplantation laboratories under Profs. John Marquis Converse and Donald L. Ballantyne on experimental skin grafts and transplantation immunity (January 1967–April 1968), reporting on his work in two seminal papers published in 1969. On Converse’s advice: “I need not emphasize to you the present trend in surgery toward a rapid evolution to transplantation research as an important part in the surgical field”, Uhlschmid had the choice to join either Prof. Martin Allgöwer, then newly appointed to the chair of visceral surgery in Basel, the renowned plastic surgeon Prof. Bengt Johanson of Gothenburg (an uncle of Dr. Uhlschmid) or Prof. Åke Senning, head of Surgery in Zurich.

Uhlschmid chose Senning at the Surgical Clinic A at the University Hospital Zurich (USZ) where he remained until his retirement in 2001, first as surgical fellow with clinical responsibilities in visceral-, lung- and transplantation surgery. His brilliant expertise and creative ideas were soon appreciated by his superiors who quickly entrusted him with the leadership of the Surgical Research Unit in addition to his clinical activities. Uhlschmid’s experimental investigations covered a wide range of problems in micro-, cryo-, laser-, and thoracic surgery with main emphasis in trachea- and kidney transplantation.

In 1973 Uhlschmid, also fluent in Swedish (his mother being a translator of Scandinavian literature), spent some time as a visitor at the Sahlgrenska University Hospital Gothenburg where he teamed up with Prof. Lars-Erik Gelin and his broad international collaborators in kidney and pancreas transplantation. Upon his return to Switzerland he joined the committee of organ conservation at Eurotransplant Leiden and established a data capturing system regarding procurement, preservation, allocation and transplantation for the USZ. After participating in the 1st Gelin-Memorial Symposium in Gothenburg in November 1981 Uhlschmid wrote in a document dated 11 April 1982 and sent to Dr. Guido Persijn: “it was felt that there was a need for a new society to be formed which would represent more accurately the aims and needs of transplantation surgery and surgeons in Europe.” Subsequently, it was Uhlschmid who was chosen to organize the “Founding Assembly Meeting” in Zurich on 28 April 1982 with Prof. Roy Calne (Cambridge) as president, Prof. Maurice Slapak (Portsmouth) as vice-president, Dr. Georg Uhlschmid (Zurich) as secretary, Prof. Walter Land (Munich) as treasurer and Profs. Hans Brynger (Gothenburg), Max Dubernard (Lyon) and Dr. Raimund Margreiter (Innsbruck) as councillors. At this meeting Prof. Heinz Pichlmaier (Köln) supported Roy Calne’s suggestion “to include *all* persons in organ transplantation, not just surgeons” and therefore, the society’s name was changed from ESTS to ESOT (European Society for Organ Transplantation). In 1982 Uhlschmid also became Swiss Citizen and got his Venia Legendi of the Zurich University with a study on new experimental methods for elongation and replacement of the thoracic trachea.

The first biannual ESOT Congress took place in Zurich in 23–25 November 1983 and was single-handedly organized by Uhlschmid. At this meeting a lot of attention was already paid to Sandimmune^®^ (cyclosporine A) which had been approved by the FDA just a few weeks before! In the aftermath—to Uhlschmid’s great disappointment—the transplant centers in Switzerland did not want to join ESOT, but instead created Swisstransplant in 4 March 1985. This prompted him to focus his research more towards general clinical and experimental visceral surgery. In 9–11 April 1984, he organized the 19th Congress of the European Society of Surgical Research (ESSR)—also in Zurich—chairing the first “Stapler Workshop” which brought him the accolade of the ESSR-presidency 1984/85.

His early investigations in reconstructive surgery in New York were now re-awakening his interest in angiogenesis and biomaterials research. His key-note contribution on “Angiogenesis—a new fascinating enigma for surgeons !?” at the first Swiss Conference on “Angiogenesis: Key Principles—Science—Technology—Medicine” in early March 1991 at St. Gallen, was highly appreciated by Judah Folkman (Harvard) and Robert Langer (MIT). A close collaboration with Prof. Ulrich Suter, then head of the Departement of Materials at the Swiss Federal Institute of Technology Zurich (ETHZ), soon resulted in two patented biopolymers for chemo-embolization, drug delivery and surgical applications (DegraPol^®^/DegraBloc^®^). Last but not least, Georg Uhlschmid encouraged many medical students to consider a career in surgery through his practical university course “Theory of surgical techniques” inaugurated in 1991 and conducted under his personal supervision. His last invited commentary at an interdisciplinary meeting at the Department of Visceral Surgery in Geneva in 26 June 2001 may be seen as his legacy with its challenging title “Surgery: evolution, revolution and entropy.” Georg K. Uhlschmid will be remembered and sadly missed by his colleagues and friends.

## Data Availability

The original contributions presented in the study are included in the article/supplementary material, further inquiries can be directed to the corresponding author.

